# Multi-Objective Power Supply Restoration in Distribution Networks Based on Graph Calculation and Information Collected by Multi-Source Sensors

**DOI:** 10.3390/s25030768

**Published:** 2025-01-27

**Authors:** Jian Dang, Shaopeng Zhang, Yile Wang, Yunjiang Yan, Rong Jia, Guangyi Liu

**Affiliations:** 1 College of Electrical Engineering, Xi’an University of Technology, Xi’an 710054, China19522511207@163.com (Y.Y.); jiarong@xaut.edu.cn (R.J.); guangyi.liu@geirina.net (G.L.); 2 Envision Digital, Redwood City, CA 94065, USA

**Keywords:** distribution grid, fault restoration, power flow calculation, graph calculation

## Abstract

With the increasing complexity of the distribution network structure, enhancing the efficiency and reliability during fault restoration has become a focal point. Based on the multi-source information collected by traditional sensors, such as CT and PT, and intelligent sensors, such as D-PMU, and the graph calculation model, the fault recovery problem of a multi-objective distribution network is studied. Firstly, a power flow calculation model and operation constraint adaptable to topology changes are proposed under the graph calculation framework. The minimum spanning tree theory is utilized to define the blackout range and recovery path set. Secondly, the intelligent sensor D-PMU is configured to collect fault information to ensure that at least one of any two connected load vertices is configured with D-PMU. Thirdly, a topological evolution model is established that considers repeated primary and secondary transfer in outage areas while exploring possible recovery strategies deeply. Finally, a distribution network in Shaanxi Province is taken as an example to verify the model. The experiment shows that the strategy in this paper dynamically adjusts the recovery strategy through four means—one transfer, one repeat transfer in the outage area, two transfers, and cutting off part of the outage load—and the overall recovery rate is increased by more than 20%.

## 1. Introduction

As the final component of the power system, the distribution network assumes a crucial role in ensuring reliable and uninterrupted power supply [1]. The quality of the user’s power supply is severely impacted when sudden faults occur. With an increasing number of distributed power sources and nonlinear loads being integrated into the distribution network, its structure and power flow become more intricate [2,3,4]. Thus, an efficient fault recovery strategy is imperative for addressing complexities in the modern power system.

Currently, mathematical optimization methods such as the branch switching method [5,6,7] and optimal power flow method [8,9,10], along with artificial intelligence algorithms like the genetic algorithm [11,12,13,14], particle swarm optimization algorithm [15,16,17,18,19], ant colony algorithm [20,21], and deep learning [22,23,24,25], are employed for distribution network path reconstruction and power supply restoration following faults. Reference [7] proposes a multi-stage power supply recovery model considering an SOP control mode and switching sequence, which can improve the power supply recovery level by coordinating switch reconfiguration, SOP control mode selection, and switching action time mapping in sequence. Reference [8] describes the dynamic reconfiguration of the distribution network as a mixed-integer, nonlinear, multi-period optimal power flow (MP-OPF) problem, whose goal is to maximize the carrying capacity of DG under thermal and voltage constraints. The application of mathematical optimization in power supply restoration was initially introduced; however, the efficacy of the employed model significantly influences the feasibility of obtaining an optimal solution while also posing challenges due to excessive computational time. Reference [13] presents a hybrid microgrid (HMG) optimal scheduling model based on an improved binary genetic algorithm (IBGA). After a power outage, IBGA can find the optimal power supply restoration scheme based on the available resources and keep the radial structure of the network unchanged. Reference [17] proposes an optimization model of distribution network fault recovery strategy considering demand response and introduces the improved particle swarm optimization algorithm based on chaos theory to optimize the mixed integer nonlinear problems. Reference [20] proposes a fault recovery strategy based on a cooperative supply of SOP and distributed power supply. The strategy sets SOP as V/f control mode. It takes the minimum weight value of the total power loss load, the minimum distributed power off the main network, and the minimum network loss as the objective function, and the optimization variables are the state of the contact switch and the outlet voltage of SOP. It then adopts the chaotic ant colony joint optimization algorithm to solve the problem. Reference [24] proposes a dynamic microgrid service restoration method based on deep reinforcement learning and treats it as a Markov decision process (MDP), which guarantees a viable radial structure of the microgrid by introducing a new method for agents to select actions while building the microgrid. Artificial intelligence algorithms, such as the genetic algorithm, have been extensively employed in addressing reconstruction problems due to their advantages in global optimization, simplicity, and efficiency. However, during population generation and hybridization variation processes, the radioactive constraints of distribution network reconstruction can lead to numerous infeasible solutions and result in local optimization. The existing fault recovery methods are briefly reviewed and summarized in Table 1.

The majority of existing studies on power supply restoration are primarily based on artificial intelligence algorithms or adjacency matrix searches for identifying restoration paths. Following distribution network faults, rapid fault location and isolation are achieved through distribution automation, while load transfer conditions facilitate the swift transfer of outage loads. However, complex active distribution grids necessitate decision-making to obtain corresponding fault restoration plans. With the increasing scale of grids and the growing number of internal power supply types, current online decision schemes struggle to meet the requirements for rapid fault restoration and fail to achieve fast, accurate, and efficient resolution of faults.

The utilization of graph databases and graph computation in power systems presents a novel approach to address this issue. For power grid data management, the graph database modeling is simple and can reflect the power grid operation status more intuitively [26,27,28,29]. Reference [30] uses graph calculation to build an evaluation model of the maximum power supply capacity of the distribution network, fully excavates the transmission capacity of the distribution network, and realizes an effective evaluation of the maximum power supply capacity of the distribution network. Reference [31] proposes a rural electrification strategy that makes use of geographic information system (GIS), graph theory, and terrain analysis to create the best electric grid topology; by using the new approach proposed, a reduction of up to 47% of the total investment cost in line deployment was achieved. Meanwhile, the combination of graph-based theory and artificial intelligence to achieve fault location in power systems has become a current research hotspot. Reference [32] proposes a fault diagnostic model for distribution systems based on deep graph learning. Reference [33] proposes a comprehensive overview of graph neural networks (GNNs) in power systems. References [34,35] propose a fault location method based on the short-time matrix pencil method (STMPM) and graph neural network (GNN), in which GNN can capture the spatiotemporal relationship between different sensor data at different locations to improve situation awareness and fault location accuracy. Reference [36] proposes a method based on unsupervised learning and graph theory to identify the four-layer topology information of a low-voltage distribution network and uses tSNE-DBSCAN-LLE algorithm to identify the four-layer topology information and generate the topology diagram. Reference [37] presents a graph theory-based topology assessment strategy (GT-ToST) to examine the limitations associated with radial structures and compare them with distribution branch reconfiguration (DBR) and the multi-stage joint distributed generation and distribution network expansion planning (DG&DNEP). Reference [38] introduces different graph theory analyses and verifies that the graph theory method can better solve the radial network optimal, low power loss, and high load voltage level of the network reconstruction problem. It can be seen that graph database and graph computing have significant advantages and some achievements in topology analysis and power flow calculation, but they have not been fully applied in reconfiguration path selection and fault restoration for the time being.

Based on this, this paper considers the multi-objective of power supply recovery and studies the fault reconstruction path and optimal recovery strategy based on graph calculation. The main contributions are as follows:

(1) A distribution grid graph model is constructed on the graph database, and a power flow calculation model with distribution grid operation constraints and adaptation to distribution grid topology changes under the graph calculation framework is also established. In addition, on the basis of traditional sensors, intelligent sensor D-PMU is rationally arranged to collect fault information.
(2) The minimum spanning tree theory is used to delineate the outage range and construct a collection of restoration paths, and the restoration strategy evaluation system is established by stratifying the power supply restoration objectives according to their correlation with the distribution grid power supply reliability, with the normal operation of the reconfigured distribution grid as the highest objective.
(3) A distribution grid topology evolution model is established to deeply explore the possible restoration strategies, determine the optimal restoration strategy according to the priority of the restoration objectives, and apply an actual distribution grid calculation case to verify the effectiveness of the proposed restoration strategy.

The remainder of this paper is organized as follows. Section 2 introduces the construction of the distribution grid graph model and the related operational constraints and tide calculations. Section 3 describes in detail the problem description and implementation method of distribution grid reconfiguration under multiple objectives, as well as the topology evolution-based power supply restoration model and restoration process. Section 4 proves the effectiveness of the method with practical cases. Section 5 concludes this paper.

## 2. Distribution Grid Operation Constraint and Power Flow Calculation Under Graph Calculation Framework

### 2.1. Construction of Distribution Grid Diagram Model

The distribution network has a natural graph structure, and its graph model is based on the graph data of the main devices in the grid as the vertex and the connection or membership relationship between the devices as the edge. Therefore, this paper constructs the distribution network graph model based on the graph database TigerGraph.

**Figure 1:**
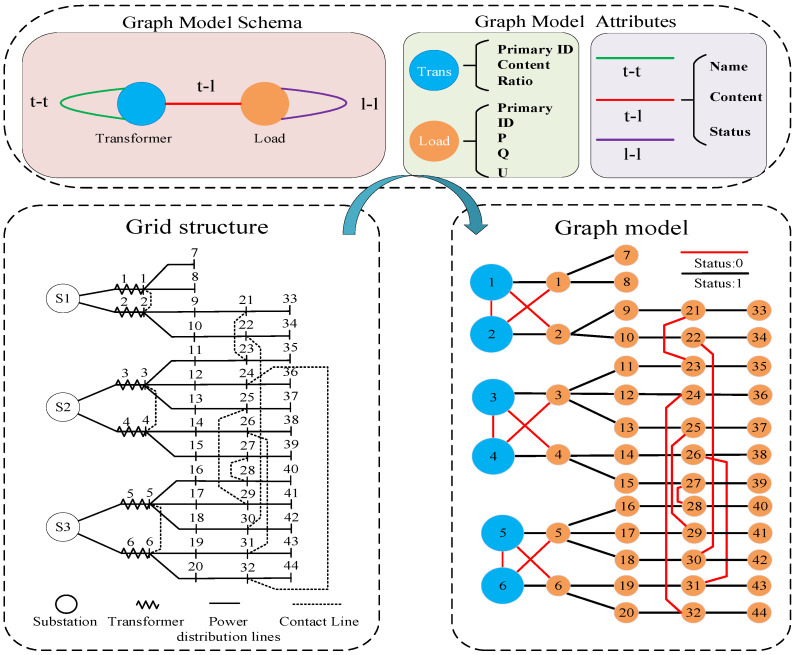
Distribution grid graph model schema.

The schema defines the vertex and edge types, connection relationships, and attributes within a graph database. To ensure an accurate and comprehensive representation of the operation status of the distribution grid, the diagram model must fully consider the interconnections between various entities in the network as well as operating information pertaining to transformers and loads. Figure 1 illustrates the modeling process for constructing the distribution network diagram model.

The graph model contains two types of vertices and three types of edges, and the vertices *Transformer* and *Load* represent the main transformer and load points, respectively. Edges *l-l* represent transmission lines and inter-station contact lines in the distribution grid, edge *t-l* represents lines between load points in the distribution grid directly to the main transformer, and edge *t-t* represents station contact switches between main transformers in the substation. The color of the edge represents the value of its *Status* attribute, with 0 being a line break and 1 being a line closure. In addition, the model also contains the respective corresponding attributes of the vertices and edges, such as the ID, capacity, and load factor of each main transformer, the ID and power of each load, and the various states of each contact line, for the subsequent calculation of the relevant diagram. In the subsequent section, *e_ij_* will be used to denote the edges connecting vertex *i* and vertex *j*.

### 2.2. Diagram Model Running Constraints

In order to ensure the proper functioning of the distribution grid and the effectiveness of fault restoration strategies, it is imperative that the following constraints be adhered to during the fault restoration process based on the distribution grid diagram model.

(1) Capacity constraint

When the distribution grid operates, it needs to meet the main transformer and line capacity constraints, and the load that can be carried on each main transformer and line should not be larger than its capacity:

(1) $$\left\{\begin{array}{l} T_{i}\leq C_{i}\\ f_{j}\leq c_{ij}\; \end{array}\right.$$

where *T_i_* is the load of the main transformer vertex *i*; *C_i_* is the capacity of main transformer *i*, i.e., the content property of main transformer vertex *I*; *f_j_* is the load passing through with load vertex *j* as the starting point; *C_ij_* is the capacity of the line upstream of load vertex *j*, i.e., the content property of edge *e_ij_*; and load vertex *i* is the upstream vertex connected to load vertex *j*.

(2) Radial constraints

Distribution network operations must meet the radial constraint; that is, each load point can only be changed from one main to its power supply. After mapping the mapping relationship to the diagram model, each load vertex has only one upstream line that is closed, so the radial constraint can be expressed as follows:

(2) $$\left\{\begin{array}{c} \sum_{i=1}^{n}\sum_{j=1}^{n}s_{ij}+\sum_{t=1}^{m}\sum_{i=1}^{n}s_{ti}=n,\;\\ \sum_{j=1}^{n}s_{ij}=1,\;\\ \sum_{t=1}^{m}s_{ti}\leq 1,\; \end{array}\right.\begin{array}{c} \forall s\in \left[0,\;1\right],\\ \begin{array}{c} {\mathrm{level}}_{i}<{\mathrm{level}}_{j},\\ i=1,\;2,\;\ldots ,\;n \end{array} \end{array}$$

where *n* and *m* are the numbers of normally operating load and primary variable vertices in the graph model; *t* is the ID of the primary variable vertex; and *level_i_* and *level_j_* are the power supply level variables of load vertices *i* and *j*, which are used to determine the upstream and downstream positions of load vertices in the topology and will be introduced in the next subsection.

(3) Voltage constraint

When the distribution grid is in normal operation, the voltage amplitude of each load point should be within the specified range:

(3) $$U_{\min}\leq U_{i}\leq U_{\max}$$

where *U_i_* is the voltage amplitude of load vertex *i*, i.e., *U* property; *U_min_* and *U_max_* are the upper and lower limits of their load vertex *i* voltage and are equal to 0.93 and 1.07, respectively.

(4) Power balance constraint

When the distribution grid is in normal operation, the power supply of each power source is equal to the sum of its load power loss:

(4) $$\left\{\begin{array}{ll} P_{t}=\sum_{t}P_{i}+\sum_{t}\Delta P_{ij}, & s_{ij}=1\\ Q_{t}=\sum_{t}Q_{i}+\sum_{t}\Delta Q_{ij}, & s_{ij}=1 \end{array}\right.$$

where *P_t_* and *Q_t_* are the active and reactive power output from the main variable vertex *t*, ∑*_t_P_i_* and ∑*_t_Q_i_* are the active and reactive load that visits vertex *i* when the main variable vertex *t* traverses down, and ∑*_t_*Δ*P_ij_* and ∑*_t_*Δ*Q_ij_* are the active and reactive power loss that visits all sides *e_ij_* when the main variable vertex *t* traverses down.

### 2.3. Power Flow Calculation in the Framework of Graphs

The graph model is capable of computing the values and properties of nodes and edges, which bears a striking resemblance to power flow calculation. Due to the unique nature of radial power supply, solving the power flow requires employing the push-back method. Within the framework of graph computation, push forward and back generation refer to breadth-first traversal processes from the end vertex forward and from the first vertex backward, respectively. Taking into account topological dynamic changes before and after reconstruction, this paper provides a comprehensive description of topology structure as well as branch node processing within the graph model. Furthermore, it proposes a reconstructed and adaptive distribution network power flow calculation model under the graph computation framework to offer data support for subsequent fault path reconstruction.

Firstly, a *visited* variable is set with the distribution grid topology unchanged, which can avoid the load vertices from being repeatedly visited in the graph model.

(5) $${\mathrm{visted}}_{i}=\left\{\begin{array}{ll} 1 & \mathrm{Vertex}\;\mathrm{is}\;\mathrm{visited}\\ 0 & \mathrm{Vertex}\;\mathrm{not}\;\mathrm{visited} \end{array}\right.$$

where *visted_i_* is the *visited* variable of load vertex *i*.

**Figure 2:**
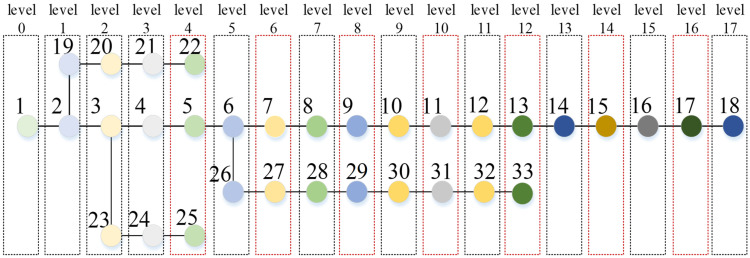
Supply level in the graph model of the IEEE 33-Bus system.

Then, each main substation in the distribution grid is modeled as a virtual load vertex, and the *Start Flag* attribute of its vertex is 1. The power supply level is set for all load vertices, which is called *level*. The level variable of the load vertex with the *Start Flag* attribute of 1 is assigned to 0, the neighboring load vertices are visited in parallel by the edge with the *Status* attribute of 1, and the *level* of the visited vertex is added to 1. After the *level* variables of all load vertices are assigned, the level variables of adjacent load vertices are compared, and if there is a load vertex *i* satisfying (6), its *End Flag* attribute is assigned to 1. Taking the IEEE 33-bus graph model as an example, the power supply levels of each load vertex are shown in Figure 2.

(6) $$\begin{array}{cc} {\mathrm{level}}_{i}>{\mathrm{level}}_{j},\; & \forall s_{ij}=1 \end{array}$$

where *level_i_* and *level_j_* are the *level* variables of load vertex *i* and load vertex *j*.

For special branch nodes in the distribution network, *usable* variables are set on all load vertices in the diagram model.

(7) $$\begin{array}{cc} {\mathrm{usable}}_{i}=\min\left({\mathrm{visited}}_{j}\right),\; & \forall s_{ij}=1\;\&\;{\mathrm{level}}_{i}<{\mathrm{level}}_{j} \end{array}$$

where *usable_i_* is the variable of load vertex *i*.

When all downstream load vertices of load vertex *i* have been visited, the *usable* load vertex *i* is 1; otherwise, it is 0.

In the process of forwarding power calculation, firstly, the upstream load vertex *visited* variable is judged to be 0; secondly, the upstream load vertex *usable* variable is judged to be 1, and the upstream load vertex can be visited only when the two constraints are satisfied at the same time to ensure the correct access of the branch load vertex; finally, the power flow calculation can be realized by adding the forward back generation method in the process of traversing the distribution grid diagram model, and the calculation process is not described here.

### 2.4. D-PMU Configuration Rules

Based on the configuration of switch position sensors on each contact switch and PT and CT on each line. In order to improve the economy and reliability of the distribution network, an intelligent sensor D-PMU can be introduced.

In the process of distribution network fault recovery, the configuration of D-PMU offers significant advantages over traditional sensors. Unlike conventional sensors, which only provide local or static data, D-PMU can synchronously acquire real-time phasor information of voltage and current and is precisely synchronized with GPS to provide high-frequency, time-consistent dynamic data. Furthermore, D-PMU can quickly provide a global view of grid state changes after a fault occurs, thereby supporting automated control systems in making intelligent recovery decisions, optimizing fault isolation and restoration paths, and significantly improving recovery efficiency and grid stability. Therefore, it is essential to establish a configuration principle for D-PMUs in the distribution network.

The configuration principle for D-PMUs is as follows: First, the first load node must be equipped with a D-PMU. Then, starting from the first load node, a D-PMU should be installed at every alternate load node. In the case of branches, a D-PMU must be installed at the starting load node of at least one branch. To ensure effective monitoring during normal operation of the distribution network, it is required that among any two connected load nodes, at least one must be equipped with a D-PMU. This configuration principle not only significantly reduces the number of D-PMUs, thereby lowering economic costs, but also ensures that during normal operation of the distribution network, voltage and current information from at least one end of each line can be measured in real-time by a D-PMU. In the distribution network topology model, if a load node *i* is not equipped with a D-PMU and is not the terminal load node of a feeder, then the other load nodes *k* and *j* connected to node *i* must be equipped with D-PMUs to ensure their electrical parameters can be effectively monitored.

Thus, in the distribution network topology model, each edge connects a load node equipped with a D-PMU and a load node not equipped with a D-PMU. To meet the global observability requirement of the distribution network, the voltage and current information of the load node without a D-PMU must be calculated based on the measurement data from the connected load node that is equipped with a D-PMU.

## 3. Multi-Objective Power Supply Restoration for Distribution Grids Based on Graph Model

### 3.1. Reconstruction Path Description Based on Minimum Spanning Tree

The radial power supply characteristics of the distribution grid ensure that the area formed by the connected load vertices of the fault outage has the same tree structure after ignoring the unclosed contact lines and fault outage lines. Based on this, the minimum spanning tree (MST) [39,40] can be used to determine the extent of fault outages in the distribution grid. In normal operation, it is equivalent to a tree with the master vertex as the root and the load vertices as the leaves. In the mode of parallel computation of vertices, the Prim algorithm with higher adaptability is selected.

The main idea of Prim’s algorithm is to traverse all the vertices starting from a certain vertex and prioritize through the edge with the lowest weight, and the tree formed after traversing all the vertices is the minimum spanning tree, which not only includes all vertices in the connected graph but also minimizes the sum of the weights of all its edges.

The downstream load vertex connected to the fault section is selected as the starting point when a fault occurs on the line. As depicted in (8), the power failure range F is determined by obtaining the minimum spanning tree by traversing the remaining load vertices using edges with a Status attribute of 1. Since the power outage region itself has a tree structure, there is no need to consider edge weights in Prim’s algorithm.

(8) $$F=F\cup i,\begin{array}{cc} & i\notin F \end{array}\;\&\;j\in F\;\&\;s_{ij}=1$$

where *F* is the set of load vertices due to fault outage, and *i* and *j* are load vertex *i* and load vertex *j*.

All load vertices in the outage region *F* are accessed in parallel, and the adjacent load vertices are accessed through the edge with *Status* attribute 0 as shown in (9), the *Reconfiguration Flag* attribute of the edge is modified to true, and the accessed load vertices are merged into the set R of reconfiguration paths.

(9) $$R=F\cup i,\begin{array}{cc} & i\notin F \end{array}\;\&\;j\in F\;\&\;s_{ij}=0$$

where *R* is the set of all load vertices on the reconfiguration path.

The set R of all load vertices on the current reconfiguration path is used as the starting point, the edges with the *Status* attribute of 1 are traversed through the load vertices as shown in (10), the *Reconfiguration Flag* attribute of the passing edges is modified to *true*, the traversal is stopped when a branch visits the virtual load vertex representing the main variable, and the resulting minimum spanning tree is the set of the reconfiguration path set.

(10) $$R=R\cup i,\begin{array}{cc} & i\notin R \end{array}\;\&\;j\in R\;\&\;s_{ij}=1$$

### 3.2. Topological Evolutionary Model

The topology evolution model is constructed based on the outage range F and the reconfiguration path set R, including the load transfer model and the load removal model.

#### 3.2.1. Load Transfer Model

In power supply restoration of distribution networks, the most basic means of power supply restoration is to detect the switch status by position sensor and realize load transfer by closing the contact line connected to the outage area. In the distribution grid diagram model, the primary transfer is as follows:

(11) sij=1size(eij)=1, Reij=true & j∈R & i∈F,j∉F

where *Re_ij_* is the Reconfiguration Flag property of edge *e_ij_*, size(*e_ij_*) is the number of edges *e_ij_*, and only one edge is modified at a time.

When closing a contact line, if the outage area is connected to two contact lines, the repeated primary transfer to the outage area can be considered. It is disconnecting one segment switch of the outage area into two parts, which is implemented in the distribution grid diagram model as follows:

(12) sij=1spq=0size(epq)=1,Reij,Repq=true & j∈R & i,p,q∈F,j∉F

The secondary transfer transfers part of the normal operating load of the transferred substation to other substations through the contact line to ensure the normal operation of the transferred substation. In the distribution grid diagram model, the secondary transfer is implemented, as shown in (13). If there is a branch of the transfer line, the transfer is given priority from the branch, and if there is no branch node, four or fewer load vertices are transferred first. The premise of secondary transfer is that the normal load to be transferred can still guarantee normal operation after being transferred; otherwise, the number of load vertices needs to be reduced.

(13) sij=1spq=0size(epq)=1,Reij=false,Repq=true & i,p,q∈R,j∉R

#### 3.2.2. Load Removal Model

If the load diversion cannot satisfy the constraint, the end load vertices need to be removed one by one and recalibrated using the power flow calculation and operation constraint model. Formula (14) is the way to remove the load in the distribution grid diagram model.

(14) $$s_{ij}=0,\begin{array}{cc} & U_{i}<U_{\min}{,\mathrm{level}}_{j}{>\mathrm{level}}_{i}\;\&\;j\in F \end{array}$$

However, according to the resection principle, in the case that there are branches in the outage area after reconfiguration and all branch end load vertices have voltages below the lower limit, two or more load vertices will be resected at one time, and the load may be resected incorrectly. For the distribution grid graph model that satisfies the operation constraint, it is necessary to judge whether the end load vertex of the reconstructed line is a branch vertex before load removal, and if it is a branch vertex, the load vertices downstream of it will be connected to the distribution grid graph model one by one and checked:

(15) $$\left\{\begin{array}{c} s_{ij}=1\\ \mathrm{size}(j)=1 \end{array}\right.,\begin{array}{cc} & \begin{array}{l} U_{i}>U_{\min}\;\&\;\mathrm{degree}(i)=3\\ \;\&\;s_{ij},s_{ik}=0\;\&\;j,k\in F \end{array} \end{array}$$

where *degree*(*i*) is the number of load vertices connected to load vertex *i* through edges with status 0 or 1, and *j* and *k* are the downstream vertex of the distribution grid reconfigured with load vertex *i*.

### 3.3. Topology Evolution-Based Power Restoration Process for Distribution Grids

#### 3.3.1. Power Supply Restoration Target Stratification

**Figure 3:**
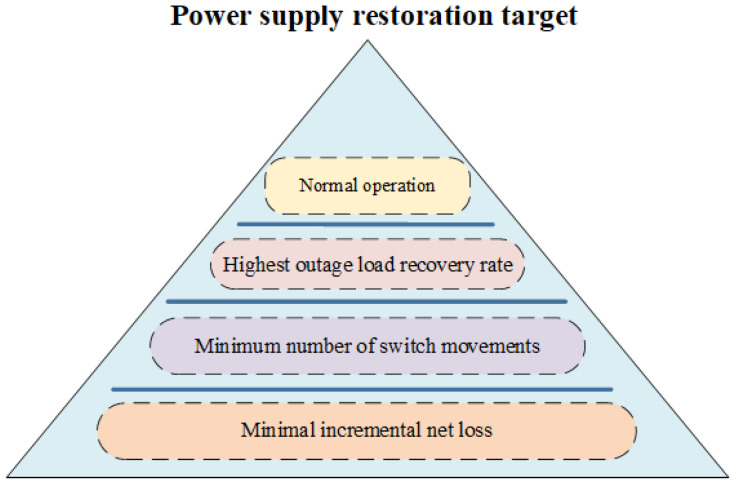
Priority power supply restoration objectives.

The objectives are stratified based on multi-objective power supply restoration, and strict priorities are determined among the objectives. There are four objectives for power supply restoration, namely “normal operation of the distribution network”, “highest outrage load recovery rate”, “minimum number of switch movements”, and “Minimal incremental net loss”. These objectives are prioritized in descending order to enhance the reliability of the distribution grid, as shown in Figure 3.

The objective of “normal operation of the distribution network” is not only the operational constraint of the reconfigured distribution grid but also the target of power supply restoration, which has the highest priority in the process of power supply restoration; the objective of “highest outrage load recovery rate” improves power supply reliability by ensuring that as many loads as possible are continuously supplied, and (16) is the recovery rate of power failure load; the objective of “minimum number of switch movements” is to improve power supply reliability by reducing operational risks, and (17) is the number of switch operations, where 1 represents the segmental switch disconnection in the faulted section. The objective of “Minimal incremental net loss” mainly considers the economy of reconstructed line operation and minimizes the improvement of power supply reliability of the distribution grid, so it is at the lowest level among the four restoration objectives. The incremental loss of reconfigured lines ΔP_LOSS_ is calculated by (18).

(16) k=∑i∈D′∩RSi∑i∈RSi

(17) $$n=\sum n_{s_{ij}0-1}+\sum n_{s_{ij}1-0}+1$$

where $\sum n_{s_{ij}0-1}$ is the number of closed contact switches in the restoration strategy, and $\sum n_{s_{ij}1-0}$ is the number of broken segment switches in the restoration strategy.

(18) $$\Delta P_{Loss}=P_{t,r}^{\prime }-P_{t,n}^{\prime }-P_{F}$$

where *t* is all the main variable vertices that supply power to the outage area after reconstruction, $P_{t,n}^{\prime }$ and $P_{t,r}^{\prime }$ are the active load supplied by the main substation vertices t before and after reconfiguration, and *P_F_* is the sum of the active load of the load vertices in the outage area after reconfiguration.

#### 3.3.2. Power Supply Restoration Process

**Figure 4:**
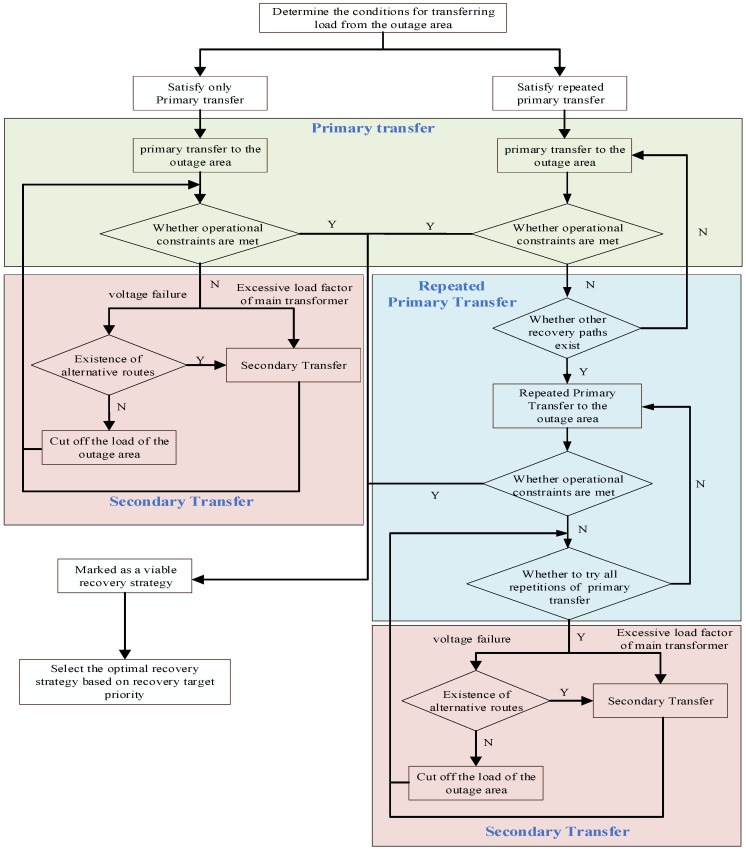
Topology evolution-based power restoration process for distribution grids.

Based on the power supply restoration objective and topology evolution model of the distribution network, the topology evolution process in the power supply restoration process of the distribution network is introduced, and the load transfer model is used to determine whether the current distribution network meets the requirements, and the process is shown in Figure 4.

The restoration strategy is optimized repeatedly by load transfer and load removal in the outage area to ensure stable operation after reconstruction. Under the condition that the highest goal of “normal operation of the distribution network” is met, the optimal recovery strategy is determined by load transfer and load removal in the outage area combined with the network loss increment of the reconstructed line. This process corresponds to the priority of the four recovery goals.

## 4. Example Analysis

**Figure 5:**
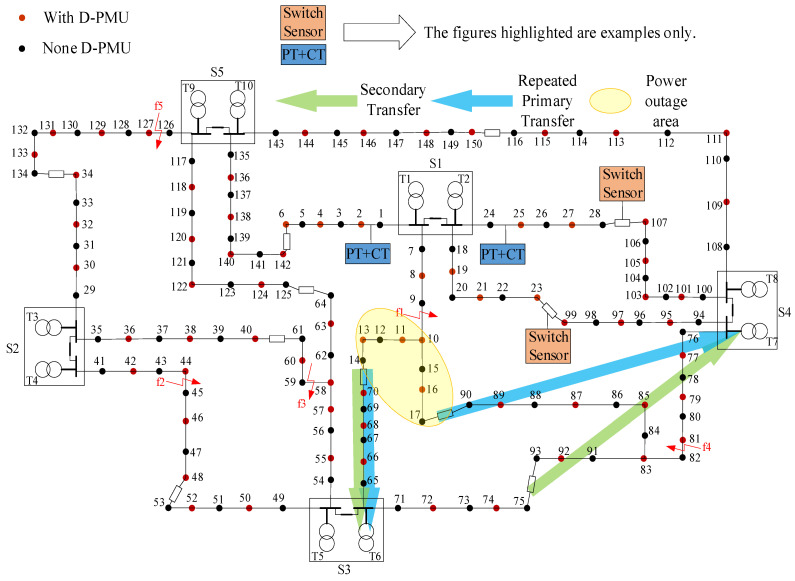
Grid voltage and current under integrated load access.

In order to verify the correctness and effectiveness of the proposed fault recovery strategy, a distribution network model with traditional sensors and intelligent sensors D-PMU was established on the TigerGraph database according to a regional distribution network in Shaanxi Province, and five faults are set, as shown in Figure 5. According to the above fault recovery process, different power supply recovery strategies are identified, and their advantages and disadvantages are evaluated.

Firstly, fault f1 is taken as an example to compare the secondary transfer with repeated primary transfer in the outage area. Based on Figure 5, it can be directly inferred that repeated primary transfer in the outage area serves two purposes: enhancing the load ratio of the switched main transformer and improving voltage at the end load vertex without impacting the original normal line power supply. Therefore, when feasible conditions are present, priority should be given to repeating power supply in the outage area.

Further analysis shows that in the scenario where a single tie line is connected to the power outage area if the power sensor detects that the transferred main transformer is overloaded, the load on the main transformer can be reduced by secondary transfer. If, after the reconfiguration, the line PT detects that the voltage at the end of the load node in the fault area is below the lower limit and there are other tie lines on the transferred line, the voltage can be increased through secondary transfer. Otherwise, only a portion of the outage load can be cut off. In case two contact lines are connected in the power outage area and an edge *e_ij_* exists within this region, if both load vertex *i* and load vertex *j*, as well as other end-load vertices in their original power outage area, fail to meet qualification criteria after disconnecting this edge, improving voltage requires closing other contact lines on diverted lines; otherwise, only part of the power outage load can be cut off.

Table 2 lists the power outage areas corresponding to the five faults, the power supply recovery strategy, and the network loss increment of the reconstructed line. Some representative recovery strategies are listed, while some recovery strategies that do not consider secondary transfer are added for comparison.

Based on the aforementioned recovery strategy, the first step involves assessing whether the reconstructed power flow calculation results of the distribution network comply with operational constraints. Amongst the five constraints in the distribution network operation model, it is primarily the variable load rate constraint and voltage constraint that render most recovery strategies impractical. In practical operations, each main transformer has a designated maximum allowable load rate, thus necessitating that their respective load rates do not exceed this threshold. Table 3 presents both normal operating load rates and maximum operating load rates for each main transformer depicted. The permissible voltage deviation for 10 kV lines within the distribution network stands at ±7% of nominal voltage, hence ensuring that neither higher than 10.7 kV nor lower than 9.3 kV are recorded as voltage amplitudes at any given load vertex. As indicated by Table 3, when calculated based on the rated voltage (10.5 kV) of each main transformer’s low-voltage side, it can be observed that no load vertex will exhibit a voltage amplitude exceeding 10.7 kV.

**Figure 6:**
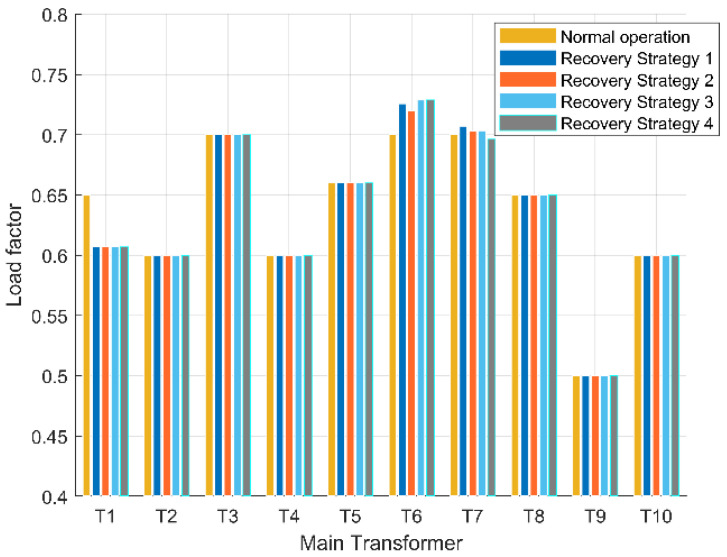
Transformer load factor in each restoration strategy of fault f1.

**Figure 7:**
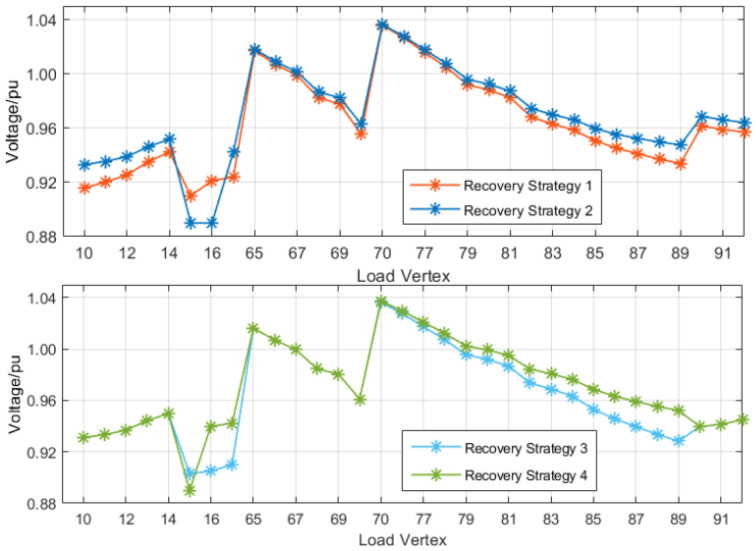
Voltage distribution in each restoration strategy for fault f1.

The reconfiguration of the distribution grid at faults f1 and f4 is discussed first. Figure 6 and Figure 7 show the voltage distribution of each load vertex within the main substation load factor and restoration path set *R* for each restoration strategy, respectively. The first two restoration strategies for fault f1 consider only the outage area repeated primary transfer, and the last two restoration strategies perform secondary transfer based on the outage area repeated primary transfer. These four strategies all satisfy the constraint in the primary load rate after reconstruction, but only restoration strategies 2 and 4 satisfy the voltage constraint, while restoration strategy 4 considers the secondary transfer, and its restoration rate is improved by more than 10% compared with restoration strategy 2, so restoration strategy 4 is the optimal restoration strategy for fault f1.

**Figure 8:**
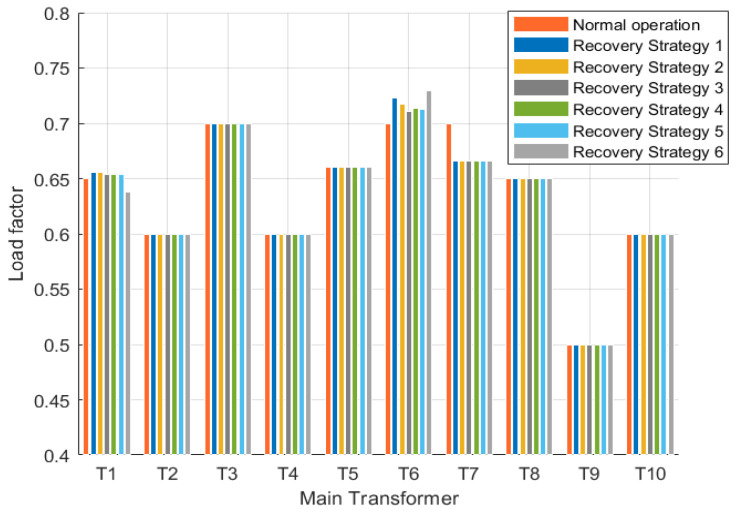
Transformer load factor in each restoration strategy of fault f4.

**Figure 9:**
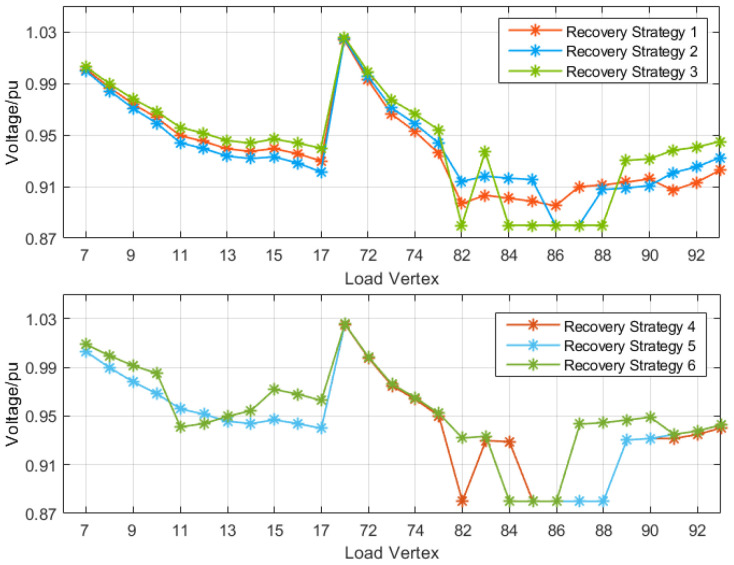
Voltage distribution in each restoration strategy for fault f4.

Fault f4 is similar to fault f1 in that the outage area also satisfies the condition of repeated primary transfer and has other contact lines on the transferred line. Figure 8 and Figure 9 show the voltage distribution of the primary variable load factor and the top point of each load in the reconfigured set R for each restoration strategy of fault f4, respectively. Six restoration strategies for fault f4 are listed in Table 2, of which the first five consider only the repeated primary transfer in the outage area, while the sixth scenario considers secondary transfer.

Among these six restoration strategies, the reconfiguration of the distribution grid using restoration strategies 3, 5, and 6 successfully meets the operational constraints. However, restoration strategy 5 results in a false cut due to load vertex 83 being a branch load vertex located at the end after reconfiguration. Restoration strategy 6 incorporates secondary transfer supply and exhibits a significantly higher restoration rate compared to strategies 3 and 5. Therefore, it can be concluded that restoration strategy 6 is deemed the optimal approach for fault f4.

By comparing the distribution grid restoration strategies for faults f1 and f4, the restoration rate of outage load is significantly higher when the secondary transfer is considered in the distribution grid restoration strategy, and secondary transfer plays a significant role in improving the reliability of the distribution grid power supply.

**Figure 10:**
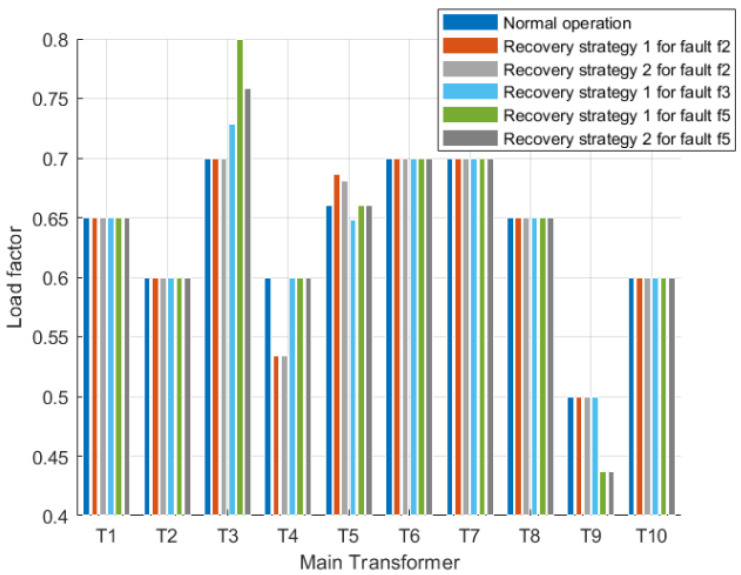
Transformer load factor in each restoration strategy of faults f2, f3, and f5.

**Figure 11:**
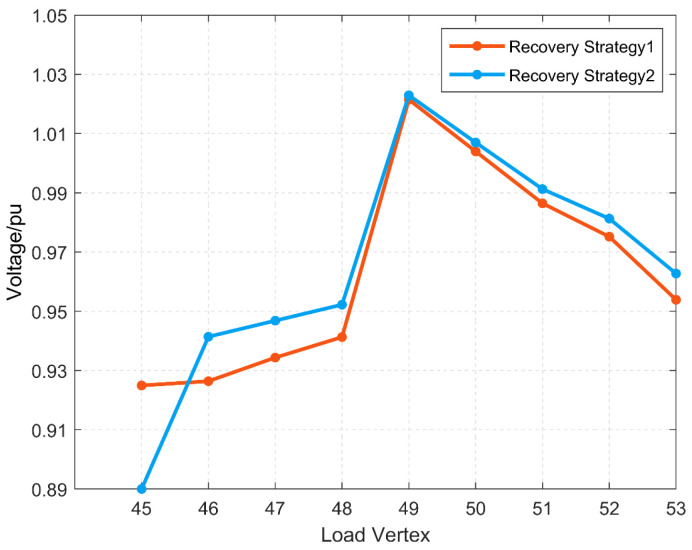
Voltage distribution in each restoration strategy for fault f2.

**Figure 12:**
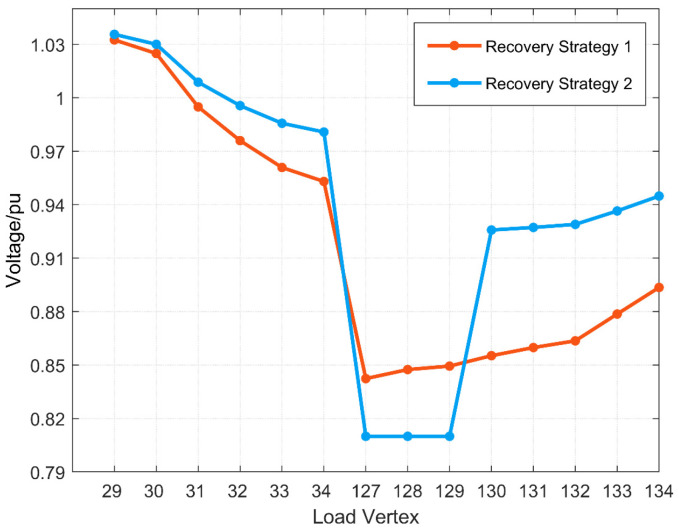
Voltage distribution in each restoration strategy for fault f5.

Figure 10 shows the main variable load ratios in each restoration strategy for faults f2, f3, and f5, and Figure 11 and Figure 12 show the voltage distribution of each load vertex in each restoration strategy for faults f2 and f5 with the reconfiguration set R, respectively. Since there is only one restoration strategy for fault f3 and the reconfigured distribution grid satisfies the voltage constraint, the voltage distribution of fault f3 is not listed separately.

For the distribution grid after the occurrence of fault f2, two restoration strategies are listed in Table 2, among which restoration strategy 1 cannot satisfy the voltage constraint, so only restoration strategy 2 can be chosen to remove the load vertex 45 located at the end of the line after reconstruction to ensure the normal operation of the distribution grid.

The fault f3 is located near the end of the original supply line, resulting in a smaller total load in the outage area and a shorter supply distance for the diverted line during normal operation. Based on this information, only one restoration strategy is recommended for fault f3, which involves closing the contact line represented by sides 40–61. This restoration strategy effectively restores power to all affected loads and ensures that the reconfigured distribution grid complies with various operational constraints.

Compared with faults f2 and f3, the location of fault f5 is close to the power source, and the reconfigured distribution grid according to the restoration strategy 1 for fault f5 not only cannot meet the voltage constraints but also the load factor of primary variable T3 has reached the maximum allowable load factor; meanwhile, the secondary transfer can reduce the load factor but cannot improve the voltage in the reconfigured outage area, so only restoration strategy 2 can be chosen to remove part of the outage load to ensure stable operation of the reconfigured stable operation of the distribution grid.

Through comparison of the examples, it can be concluded that the distribution network power supply restoration model proposed in this paper can strictly follow the reconstruction target excellence level to obtain the optimal recovery strategy and has good adaptability to the topological changes of the distribution network. When the reconstructed distribution network does not meet the operation constraints, priority should be given to improving the operating conditions of the reconstructed distribution network by repeating primary or secondary transfers in the outage area. When cutting the outage load, consideration should be given to the special situation that there are branches at the end so as to avoid cutting the load that should not be cut.

## 5. Conclusions

This paper conducts research on multi-objective power supply restoration in distribution networks based on graph calculation, leveraging the inherent consistency between the distribution network diagram model and its topological multi-source information. The primary contributions are as follows:

(1) The power flow calculation model for power distribution networks is proposed, and the configuration rules of intelligent sensor D-PMU are formulated to ensure that at least one of every two nodes is configured with a sensor within the framework of graph theory. Real-time generation of power supply levels for each load vertex ensures adaptability to changes in the distribution network topology.
(2) By considering the radial power supply constraint, the minimum spanning tree method and line PT, CT, and other sensors are used to collect fault information and determine the fault outage area, and the reconstruction path set is established. A stratified objective for power supply restoration is defined, with the ultimate goal being the normal operation of the distribution network after reconstruction.
(3) An evaluation system for restoration strategies is developed. A topological evolution-based power supply restoration model is established, where all possible restoration strategies are explored through topological evolution, and an optimal strategy is determined based on priority. The calculation results show that the recovery strategy with a 100% recovery rate and minimum network loss can be guaranteed by comparing the constraints under five kinds of faults.

In the follow-up study, we should consider the role and influence of distributed power supply in the graph model, the action sequence of switches during fault recovery, and the realization of automatic control of sensors in fault recovery.

## Figures and Tables

**Table 1:** Summary of existing fault recovery methods.

Refs.	Method Used	Targeted Problem	Improved Model
[5,6,7]	Branch switching	Active reconstruction	The two-stage robust optimization model
		Power supply restoration	The two-stage recovery strategy for concurrent faults
		Power supply restoration	The multi-stage supply restoration method of ADNs
[8,9,10]	Optimal power flow	Active reconstruction	The multi-period optimal power flow
		Power supply restoration	Distribution automation system and the real-time control capability of EDC
		Power supply restoration	The mixed-integer second-order cone programming
[11,12,13,14]	Genetic algorithm	Active reconstruction	The genetic algorithm based on all spanning trees of undirected graph
		Power supply restoration	The Dijkstra algorithm and genetic algorithm
		Active reconstruction	The improved binary genetic algorithm
		Active reconstruction	Change the equivalent impedance of the common connection point of the inverter
[15,16,17,18,19]	Particle swarm optimization algorithm	Power supply restoration	The preference multi-objective particle swarm algorithm considering the reference vector
		Active reconstruction	The multi-period, multi-objective distribution expansion planning model
		Power supply restoration	The improved particle swarm optimization algorithm based on the chaos theory
		Power supply restoration	The binary particle swarm optimization
[20,21]	Ant colony algorithm	Power supply restoration	The service restoration strategy by cooperating SOPs with distributed generators
		Active reconstruction	The methodology for placement of sectionalizing switches in distribution networks
[22,23,24,25]	Deep learning	Active reconstruction	The Branching Dueling Q-network
		Power supply restoration	The robustness enhancement method for DRL-enabled distribution system load restoration
		Power supply restoration	The novel restoration method using Markov decision process
This paper	Graph calculation based on minimum spanning tree	Power supply restoration	The distribution grid topology evolution model

**Table 2:** Comparison of distribution grid restoration strategies.

Fault	Outage Area	Restoration Strategy				Reconfigure Line Grid Loss Increment/kW
		NO.	Switch Operation	Outage Load Top	Restoration Rate	
f1	10, 11, 12, 13, 14, 15, 16, 17	1	disconnect 15-16; close 14-70, 17-90	\	100%	3.97
		2	disconnect 10-15, 16-17; close 14-70, 17-90	15, 16	71.83%	3.44
		3	disconnect 10-15, 83-91; close 14-70, 17-90, 75-93	\	100%	7.02
		4	disconnect 10-15, 15, 16, 83-91; close 14-70, 17-90, 75-93	15	81.86%	6.87
f2	45, 46, 47, 48	1	close 48-53	\	100%	3.27
		2	disconnect 45-46; close 48-53	45	78.94%	2.96
f3	59, 60, 61	1	close 40-61	\	100%	1.52
f4	82, 83, 84, 85, 86, 87, 88, 89, 90, 91, 92, 93	1	disconnect 86-87; close 17-90, 75-93	\	100%	4.79
		2	disconnect 85-86, 87-88; close 17-90, 75-93	86, 87	82.02%	4.58
		3	disconnect 82-83, 83-84, 88-89; close 17-90, 75-93	82, 84, 85, 86, 87, 88	45.25%	3.98
		4	disconnect 82-83, 84-85, 88-89; close 17-90, 75-93	82, 85, 86, 87, 88	62.08%	4.06
		5	disconnect 83-84, 88-89; close 17-90, 75-93	84, 85, 86, 87, 88	57.34%	4.09
		6	disconnect 10-11, 83-84, 86-87; close 14-70, 17-90, 75-93	84, 85, 86	71.06%	6.00
f5	127, 128, 129, 130, 131, 133, 134	1	close 34-134	\	100%	2.13
		2	disconnect 129-130 close 34-134	127, 128, 129	59.29%	1.53

**Table 3:** Transformer parameters.

NO.	Content/MAV	Ratio/(kV·kV^−1^)	Operating Load Factor	Allowable Load Factor
T1	40	110/10.5	0.65	0.8
T2	40	110/10.5	0.6	0.8
T3	20	35/10.5	0.7	0.8
T4	20	35/10.5	0.6	0.8
T5	50	110/10.5	0.66	0.85
T6	50	110/10.5	0.7	0.85
T7	40	110/10.5	0.7	0.8
T8	40	110/10.5	0.65	0.8
T9	31.5	35/10.5	0.5	0.85
T10	31.5	35/10.5	0.6	0.85

## Data Availability

Data are contained within the article.
